# Analysis of aortic valve prostheses using advanced cardiovascular imaging—a patient-specific reversed translational approach

**DOI:** 10.1038/s41598-026-44295-w

**Published:** 2026-03-18

**Authors:** Linda Grefen, Christopher Herz, Jana Flexeder, Maximilian Grab, Christoph Mueller, Sven Peterss, Caroline Radner, Joscha Buech, Sebastian Sadoni, Dirk-André Clevert, Daniel Giese, Christian Hagl, Nicola Fink, Adrian Curta

**Affiliations:** 1 Department of Cardiac Surgery, LMU University Hospital, LMU Munich, Munich, Germany; 2 DZHK (German Centre for Cardiovascular Research), partner site Munich Heart Alliance, Munich, Germany; 3 Chair of Medical Materials and Implants, Technical University Munich, Munich, Germany; 4 Department of Radiology, LMU University Hospital, LMU Munich, Munich, Germany; 5 Magnetic Resonance, Siemens Healthineers AG, Erlangen, Germany

**Keywords:** TAVR, Rapid-deployment, 3D Printing, 4D Flow MRI, Ultrasound, Cardiology, Diseases, Engineering, Medical research

## Abstract

Rapid-deployment and transcatheter aortic valve replacement (RDAVR and TAVR) are standard treatment options for aortic valve disease. The lack of uniformity in size labelling and potentially adverse hemodynamic effects emphasize the need for an in-depth comparison of available prostheses. State-of-the-art valves (INTUITY Elite, Perceval, SAPIEN 3 Ultra and Evolut PRO+) were implanted into a patient-specific 3D printed aortic phantom with an annulus size recommended for the evaluated valves. The model was incorporated into a flow loop setup. Analysis of hemodynamic parameters was performed using advanced cardiovascular imaging (4D flow MRI and vector (V) flow ultrasound (US)). US revealed systematic differences in flow rates between RDAVR and TAVR prostheses. 4D flow analysis revealed localized regions of elevated WSS heterogeneously distributed without relevant differences between the prostheses. RDAVR prostheses showed higher kinetic energy loss (EL) along the thoracic aorta in small- compared to large-diameter models. EOA estimations ranged from 2.02 to 2.69 cm^2^ for RDAVR to 1.71 cm^2^ for 23 mm TAVR. Pressure gradients varied strongly between prosthesis types. Blood flow characteristics presented notable differences between the considered prostheses. This interdisciplinary approach provides a foundation for interpreting clinical outcomes after TAVR or RDAVR and potentially reduce the risk of prosthesis-patient mismatch.

**Supplementary Information:**

The online version contains supplementary material available at 10.1038/s41598-026-44295-w.

## Background

Aortic valve (AV) replacement is one of the most frequently performed procedures in cardiac surgery and interventional cardiology. Biological heart valve prostheses can be subdivided into prostheses for catheter-based heart valve replacement (TAVR, transcatheter aortic valve replacement) and conventional surgical prostheses including rapid-deployment aortic valve prostheses (RDAVR, rapid-deployment aortic valve replacement)^1^. Use of the latter is intended to combine the advantages of open surgical (complete removal of the degenerated AV and controlled implantation under visualization) and catheter-based AV replacement (less invasive and faster implantation)^2,3^.

Pre-operative planning of an AV replacement is crucial, and the selection of intervention follows various guidelines^4^. The selected valve size is, however, usually solely based on the annulus diameter and the corresponding manufacturers’ recommended labelled size of each prosthesis. Differences in hemodynamic performance of valve prostheses as well as the influence of patient-specific anatomical conditions are rarely considered^5,6^. Anomalies in the movement and thickness of the leaflets, as well as their orientation in relation to the ascending aorta (AscAo) defined by the specific design, implantation level, and angle of the prosthesis further influence aortic hemodynamics^7,8^. These parameters can have a tremendous impact on prosthesis-patient-mismatch which is knowingly associated with poorer long-term results and even higher mortality^9,10^.

4D flow Magnetic Resonance Imaging (MRI) is an established method for assessing aortic hemodynamics^11^. It allows for both, visualization of blood flow and retrospective quantification of velocity and flow rate parameters at any location within a defined volume^12^. Compared to experimental techniques, e.g. stereo- or tomographic particle image velocimetry (PIV), 4D flow MRI enables accurate scanning of great vessel geometries in a time-resolved manner^13,14^. Additionally, vector (V) flow ultrasound (US) imaging, with its ability to detect the true vector velocity in complex, non-laminar flow regimens, allows for real-time collection of empirical data and flow analysis^15,16^.

The aim of the present study was to investigate potentially adverse hemodynamic effects beyond annular size and corresponding manufacturers’ recommendation of labelled size and to emphasize a comprehensive comparison of commercially available RDAVR and TAVR prostheses. Hence, hemodynamic parameters within a 3D printed patient-specific aortic phantom were analyzed and compared using four state-of-the-art heart valve prostheses. The aortic phantom was integrated in a dedicated in vitro mock circulatory flow loop. The evaluated prostheses are, according to manufacturers’ size specifications, intended for implantation in the herein used models’ aortic annulus size. Qualitative and quantitative assessment was achieved utilizing complementary V flow US and 4D flow MRI.

## Materials and methods

### In vitro setup and experimental conditions

**Fig. 1:**
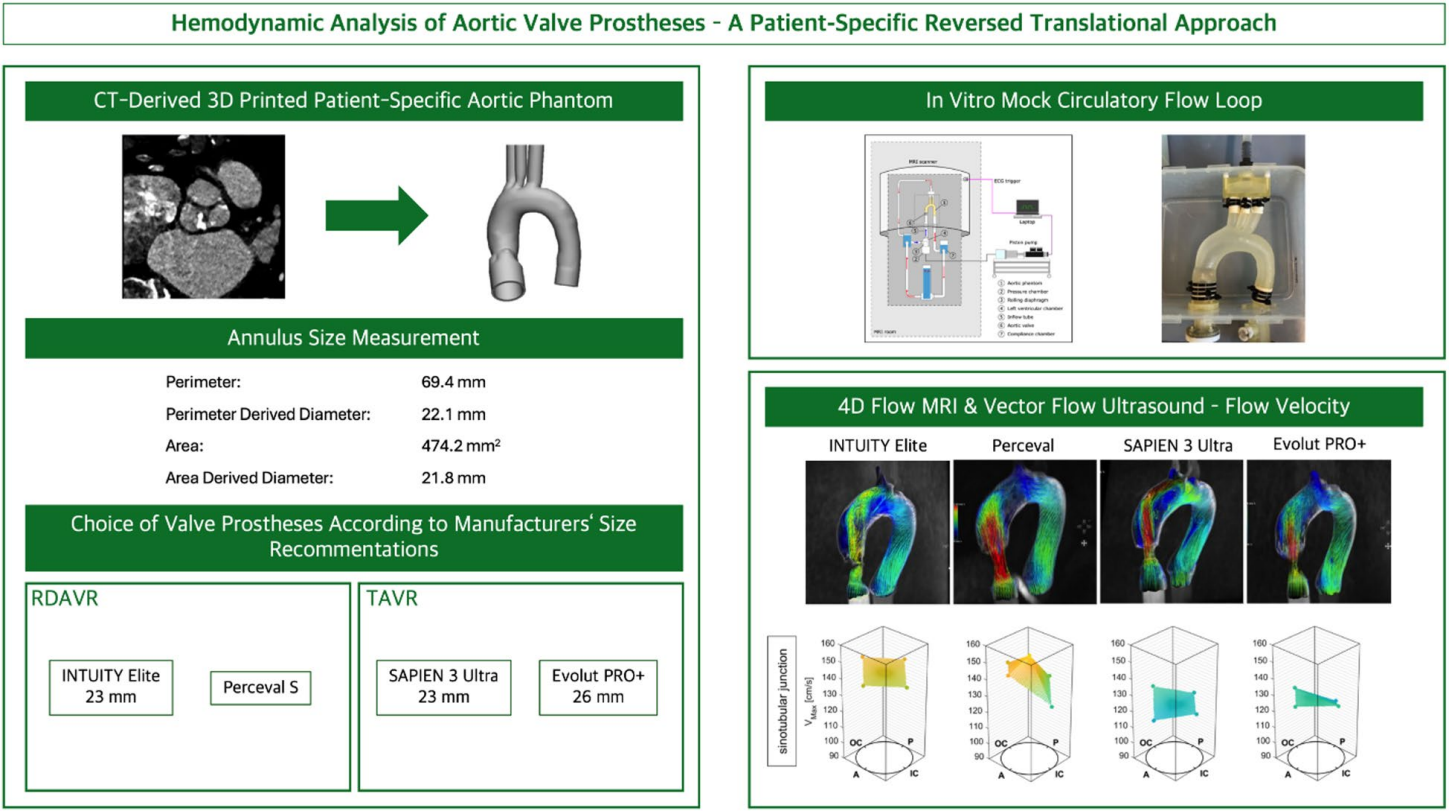
Overview of experimental setup. Figure created with^17,25^.

The aortic models were based on an anonymized pre-operative CT dataset of a female patient with small annular diameter, planned for AV replacement (Fig. 1, overview). The use of the contrast-enhanced CT dataset was approved by the Ethics Committee of the Ludwig Maximilians University (18–188 UE), informed consent was obtained and working with patient-specific data was carried out in accordance with relevant guidelines and regulations. Measurements were performed using 3mensio (3mensio Medical Imaging B.V., Structural Heart, v. 10.7, Utrecht, Netherland^17^ resulting in an average aortic annulus of 22.3 mm (min = 20.3 mm, max = 24.3 mm) and a perimeter of 69.4 mm. To create the digital 3D model, the contrast-enhanced CT dataset was imported to Mimics Innovation Suite (Materialise Mimics 25.0, Materialise NV, Leuven, Belgium). Subsequently, the region of interest was segmented, and the calculated part was imported in 3-matic (Materialise 3-matic 17.0, Materialise NV) for mesh optimization and design adaptation steps. To manufacture the models, a Polyjet printer (Agilista 3200 W, Keyence Co., Osaka, Japan) was used with a flexible material (AR-G1L, Keyence Co.) for the models and a water-soluble material (AR-S1, Keyence Co.) as support material. Following the post-processing steps, the prostheses were implanted within the aortic annulus in accordance with the manufacturers’ instructions for the use of each device. This step was performed by the heart team of the LMU University Hospital to ensure the correct valve positioning within the aortic root (AoRoot). The following prostheses were selected based on the manufacturers’ labelling, ensuring compatibility with the chosen aortic annulus dimensions obtained from the CT dataset: INTUITY Elite 23 mm (Edwards Lifesciences, Irvine CA, USA), Perceval S (LivaNova, London, United Kingdom), SAPIEN 3 Ultra 23 mm (Edwards Lifesciences), and Evolut PRO + 26 mm (Medtronic, Minneapolis, MN, USA).

The experimental setup simulated a pulsatile flow through the thoracic aorta as previously described (Supplement 1)^18^. The setup’s simulated parameters were based on the specifications of DIN EN ISO 5840 for in vitro analysis of surgically implanted heart valve substitutes and heart valves implanted by transcatheter technique. A physiological mean pressure of 100 mmHg was kept constant throughout the image acquisition. The cardiac output was set to 5.0 l/min and the heart rate to 70 BPM. The systolic duration was set to 35%. A 40% (v/v) glycerine/water solution served as a blood substitute, replicating blood viscosity as suggested previously^19,20^.

### Radiological imaging acquisition and analysis

Localized values for flow velocities and wall shear stress (WSS) in the aortic model were determined using a state-of-the-art sonographic device (Resona R9, Mindray Medical Int. Ltd., Shenzhen, China) and a linear array probe (L11-3U, Mindray Medical Int. Ltd.). Imaging was performed using the V flow mode, a dedicated technique previously described by Jensen et al., commercially available on Mindray Resona R9 systems^21,22^. Flow velocities were measured at three analysis planes positioned perpendicular to the vessel’s centerline at the following locations: sinotubular junction (STJ), mid-ascending aorta (midAoAsc), and distal ascending aorta (disAoAsc) (Supplement 2). In each plane, the flow rate was measured within five regions of interest (ROI) positioned at the vessel’s center, as well as the anterior and posterior walls, and the inner and outer curvatures of the vessel. Values were averaged over *n* = 5 measurements. The WSS was determined near the anterior, inner curvature, posterior, and outer curvature vessel wall. The values were averaged over five points within the ROI for *n* = 5 measurements.

MRI was performed using a 1.5 Tesla scanner (MAGNETOM Avanto Fit, Siemens Healthineers, Erlangen, Germany) and a research sequence. The methodological approach to acquire the radiological images and perform the post-hoc analysis in cvi42 imaging software (cvi42 version 5.17.2, Circle Cardiovascular Imaging Inc., Calgary, Canada^23^) was analogous to previous studies^18,24^. The MRI data was evaluated at pre-defined planes located between the STJ and the AoDesc (Supplement 2). The maximum and mean values of the measured parameters refer to the cardiac cycle averaged over the entire image acquisition. The effective orifice area (EOA) was quantitatively determined in cvi42. Visualization of the flow velocity in a cross-sectional plane, identified as peak velocity position downstream of the AV and thus *vena contracta*, was used to assess the qualitative position and shape of the EOA.

### Statistics

The statistical analysis was performed using GraphPad Prism v9.3.0 (GraphPad Software, San Diego, USA). 3D plots were created using MATLAB (MATLAB 9.14 (R2024a), The MathWorks Inc., Natick, Massachusetts, US^25^). Continuous variables are expressed as mean ± standard deviation. Statistical t-tests were performed to compare the flow velocity and WSS measurements for the valves considered. A *p* value < 0.05 was considered statistically significant and a post-hoc Bonferroni correction was applied.

## Results

### Flow velocity

**Fig. 2:**
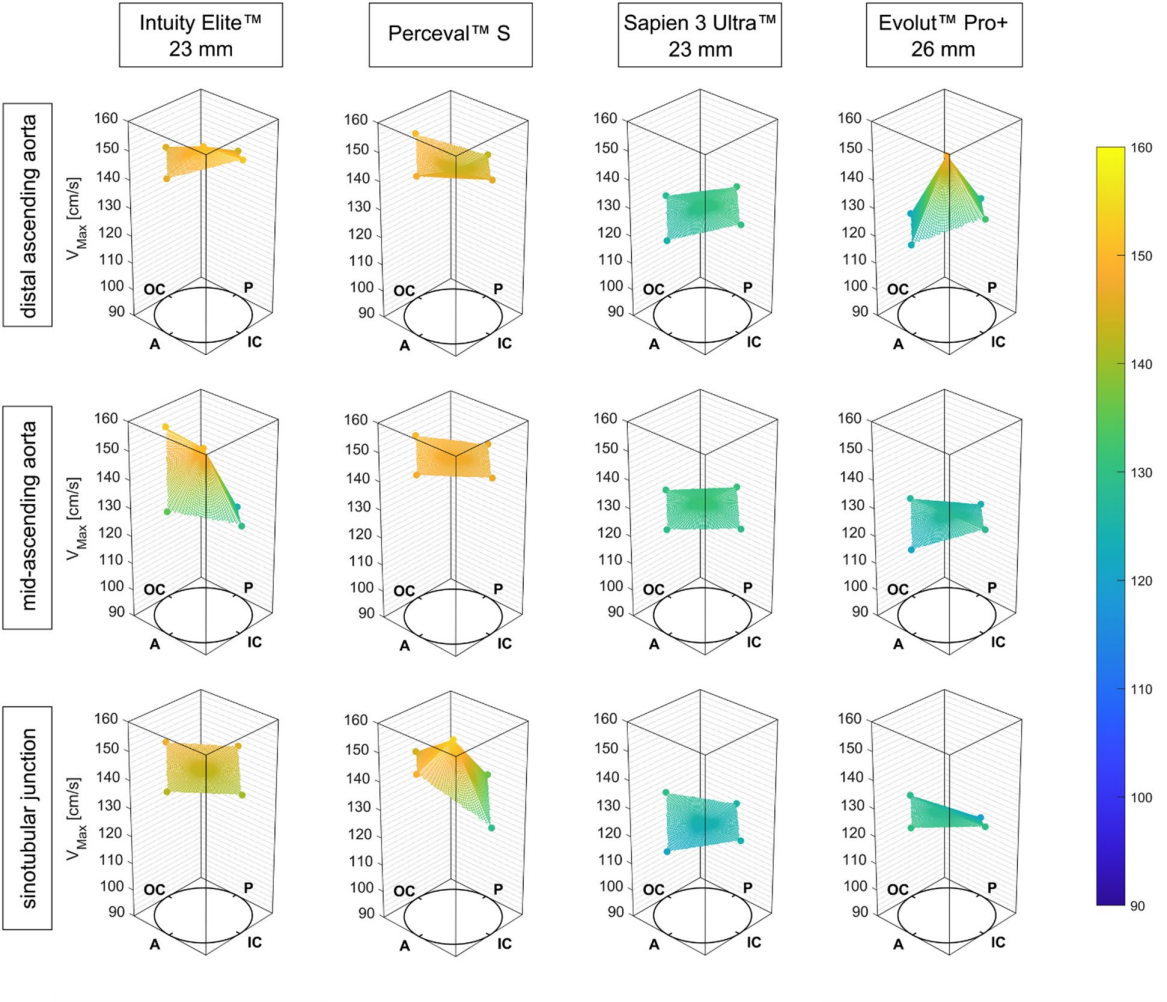
Flow velocities measured using vector flow ultrasound at the center of the vessel and at four near-wall regions of interest. The mean values are connected by a 3D mesh-grid to illustrate the distribution of flow velocity at the analysis plane. The mesh-grid color indicates the magnitude within the presented scale. *A* anterior, *IC* inner curvature, *P* posterior, *OC* outer curvature.

Mean values for flow velocity determined using V flow US are shown in Fig. 2. In general, the recorded and averaged flow velocities of the TAVR valves were significantly lower than those of the RDAVR valves. Only the comparison of velocities at the midAoAsc for the INTUITY 23 mm and SAPIEN 3 Ultra 23 mm prostheses missed statistical significance (*p* = 0.16). Throughout both groups, maximum flow velocity at the STJ was measured within the cantered ROI for the Perceval S valve (154.13 ± 2.73 cm/s). In contrast, the maximum flow velocity associated with the INTUITY 23 mm was measured along the lateral portion (i.e. outer curvature) of the midAoAsc (152.6 ± 4.87 cm/s). Flow velocities for the considered TAVR valves were comparable at all analysis planes. Only the maximum velocity at the center of the disAoAsc for the Evolut PRO + was significantly higher compared to the SAPIEN 3 Ultra, with 148.07 ± 4.99 and 130.43 ± 1.17 cm/s, respectively (*p* < 0.001, Table 1).

**Table 1:** Hemodynamic parameters at the sinotubular junction obtained in 4D flow Magnetic Resonance Imaging.

	INTUITY Elite 23 mm	Perceval S	SAPIEN 3 Ultra 23 mm	Evolut PRO + 26 mm
V_max_ [cm/s]	202.18	254.29	216.38	190.22
EOA [cm^2^]	2.235	2.019	1.705	2.464
ΔP [mmHg]	6.67	8.48	8.88	10.90
EL [µW/mm^3^]	1.85	7.71	2.72	3.40

*V*_*max*_ maximum flow velocity, *EOA* effective orifice area, *ΔP* pressure gradient, *EL* kinetic energy loss.

Flow velocities recorded and averaged over one cardiac cycle were generally higher compared to V flow US measurements. Consistent with the V flow US measurements, flow velocities for the Perceval S valve showed highest values with 254.29 cm/s. The Evolut PRO+ valve yielded the lowest flow velocity with 190.22 cm/s.

### Wall shear stress

**Fig. 3:**
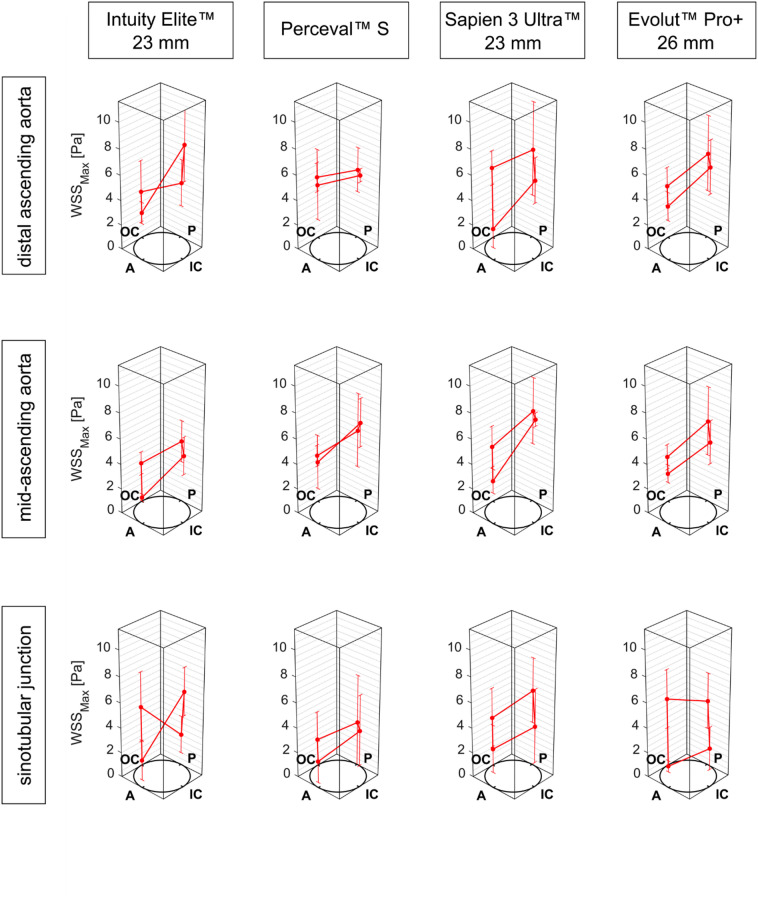
Distribution of the maximum wall shear stress (WSS_Max_) assessed with vector flow ultrasound. The values are shown as mean ± standard deviation for *n* = 5 measurements within four regions of interest at defined analysis planes. *A* anterior, *IC* inner curvature, *P* posterior, *OC* outer curvature.

V flow US revealed detailed insight into WSS distribution at the STJ, midAoAsc, and disAoAsc (Fig. 3). Maximum WSS (WSS_Max_) was subject to high fluctuations. When comparing potential prosthesis-associated reductions or elevations in WSS_Max_ within the defined ROI at each measurement plane along the vessel centerline, only a few significant differences between individual models could be determined. At the STJ along the inner curvature, WSS_Max_ determined for the INTUITY 23 mm was significantly higher compared to Evolut PRO+, at 7.56 ± 1.83 Pa and 3.14 ± 1.71 Pa, respectively (*p* = 0.003). The most pronounced differences between the valve-associated values for WSS_Max_ were found between the Perceval S and both TAVR protheses. The calculated WSS_Max_ within the lateral ROI at the STJ was significantly higher for the Evolut PRO+ compared to the Perceval™ S (5.46 ± 2.31 Pa and 2.16 ± 2.26 Pa, *p* = 0.05), while WSS_Max_ measured along the anterior portion of the disAoAsc was significantly lower for the SAPIEN 3 Ultra than the Perceval S (2.56 ± 1.53 Pa and 6.02 ± 1.40 Pa, *p* = 0.04).

**Fig. 4:**
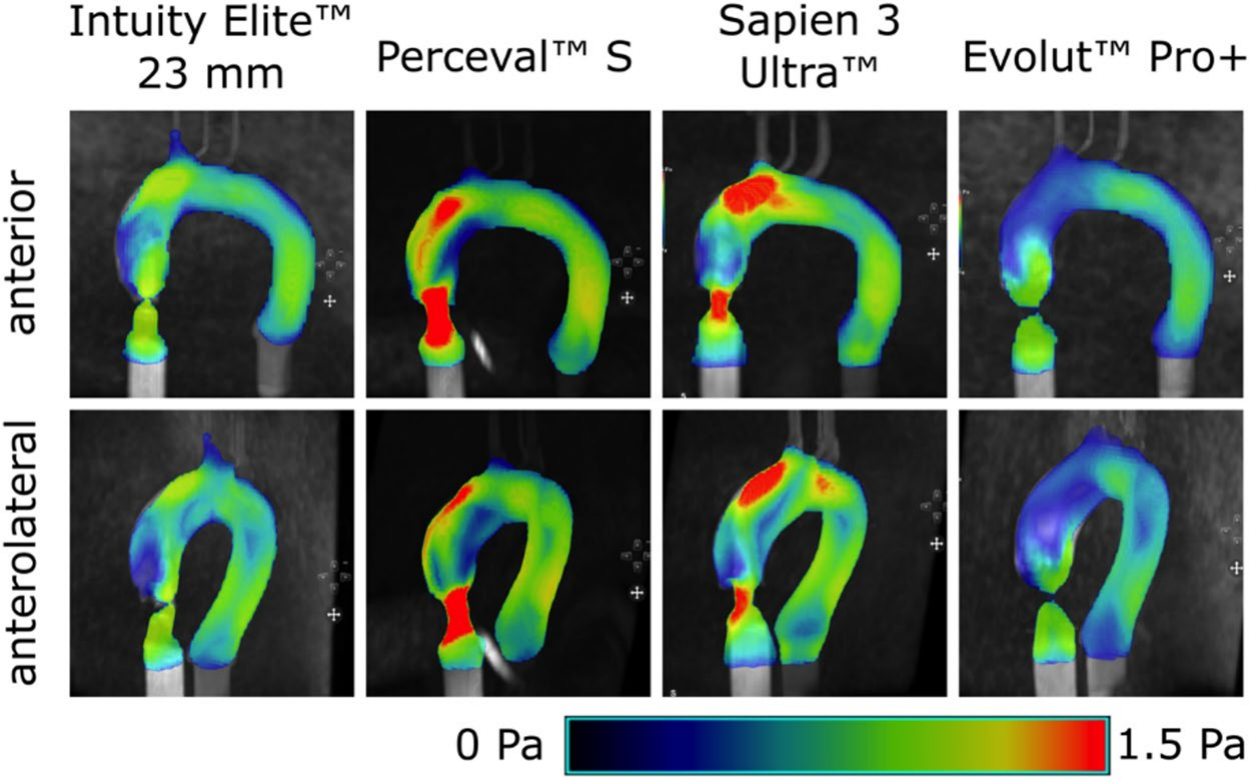
Qualitative wall shear stress distribution during peak systole of the cardiac cycle visualized in anterior and anterolateral view in 4D flow Magnetic Resonance Imaging.

The qualitative WSS distribution was obtained by visualization of the 4D flow MRI data and is shown in Fig. 4. Areas of high WSS were heterogeneously distributed. Localized regions of elevated WSS were found at the AoRoot, the outer curvature of the disAoAsc, the anterior aortic arch (AoArc), and the anterior and posterior portion of the descending aorta (AoDesc). There were no relevant regional differences between the TAVR and RDAVR valves. In general, WSS was lowest for the INTUITY 23 mm and Evolut PRO+ valves.

WSS at the level of the STJ was inversely proportional to the prosthesis size considering both TAVR models. SAPIEN 3 Ultra yielded the highest WSS with 0.54 Pa, while WSS for the Evolut PRO + was found at 0.18 Pa (Supplement 3).

### Pressure gradient

**Fig. 5:**
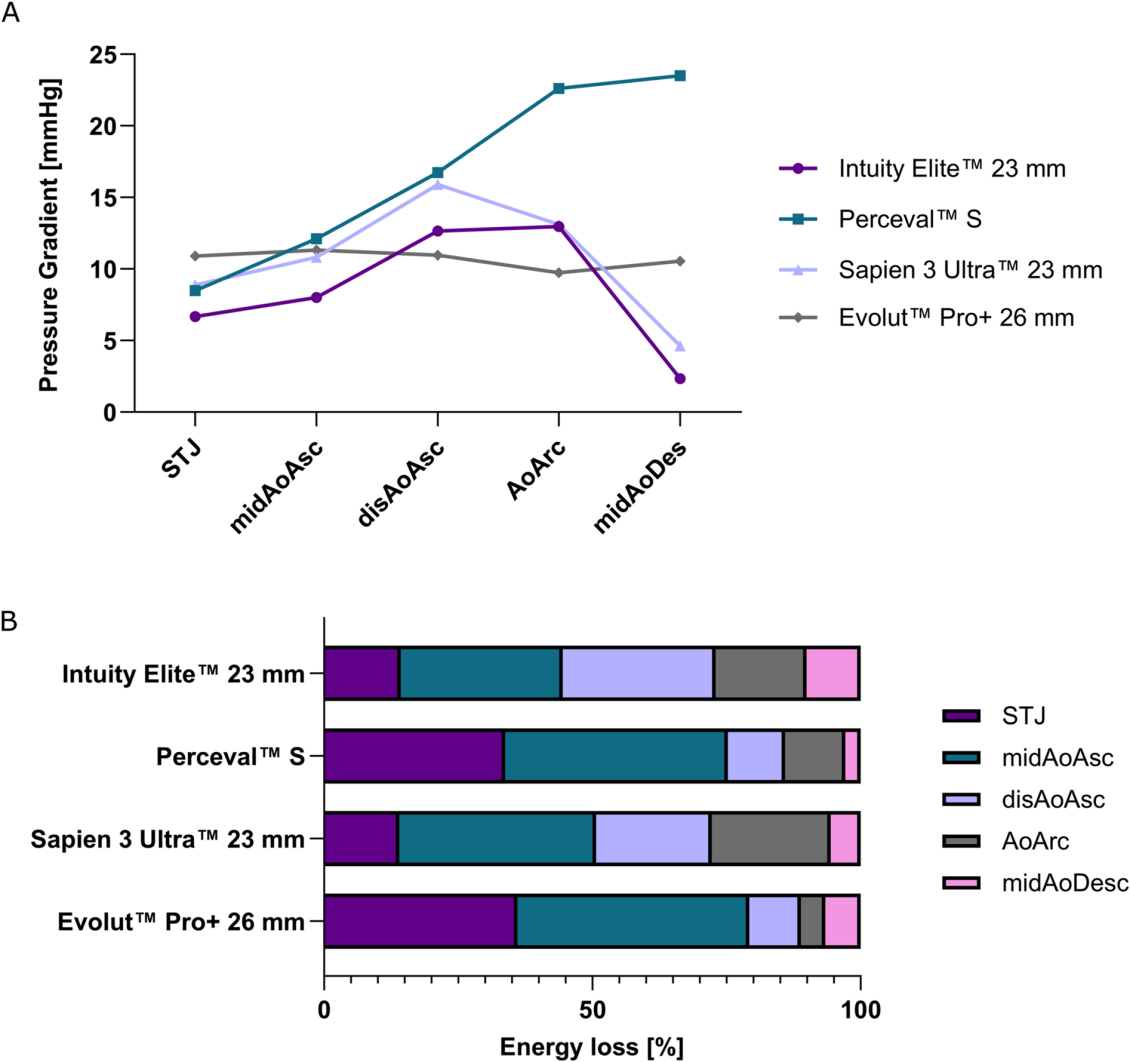
Quantitative assessment of the pressure gradient and kinetic energy loss (EL) using 4D flow Magnetic Resonance Imaging: (**A**) Pressure gradient at distinct locations within the aortic phantom with respect to the reference analysis plane coinciding with the heart valve, (**B**) Comparison of the portions of total kinetic EL, i.e. relative kinetic EL between the defined analysis planes for all valves considered. *STJ* sinotubular junction, *midAoAsc* mid-ascending aorta, *disAoAsc* distal ascending aorta, *AoArc* aortic arch, *midAoDesc* mid-descending aorta.

The pressure gradients with respect to the distinct reference plane positioned proximal to the heart valves are shown in Fig. 5A. The INTUITY 23 mm and SAPIEN 3 Ultra valves exhibited similar values throughout the entire model. Values in the section confined by the brachiocephalic trunk and the left subclavian artery, as well as within the mid-descending aorta were substantially higher for the Perceval S compared to the other valves, with maximal pressure gradients reaching as high as 23.5 mmHg. Of note, the Evolut PRO+ valve did only yield values in the range between 9.73 and 11.33 mmHg.

### Kinetic energy loss

The Perceval S valve was characterized by higher values of kinetic EL throughout all analysis planes. Absolute kinetic EL measured between the reference plane positioned caudal to the valve and the analysis plane located at the STJ was almost four times higher compared to the INTUITY 23 mm valve with 7.71 and 1.85 µW/mm^3^, respectively. In comparison, absolute kinetic EL at the STJ were found at 2.72 and 3.40 µW/mm^3^ for the SAPIEN 3 Ultra and Evolut PRO+ prostheses. The latter was associated with the lowest absolute kinetic EL up to the mid-descending aorta (midAoDesc) with only 9.44 µW/mm^3^ (Supplement 4).

The evaluation of portions of the total kinetic EL along the aortic phantom’s centerline showed no distinct difference between the RDAVR and TAVR group (Fig. 5B). Despite their considerable difference in label size, the relative distribution of kinetic EL was very similar for the Perceval S and Evolut PRO + 26 mm valves for the analysis planes positioned at the STJ, midAoAsc, and disAoAsc. In contrast, the 23 mm RDAVR and TAVR prostheses presented comparable values for relative kinetic EL at the STJ (14.29 and 14.00%) and therefore markedly lower compared to two other valves considered.

### Effective orifice area

**Fig. 6:**
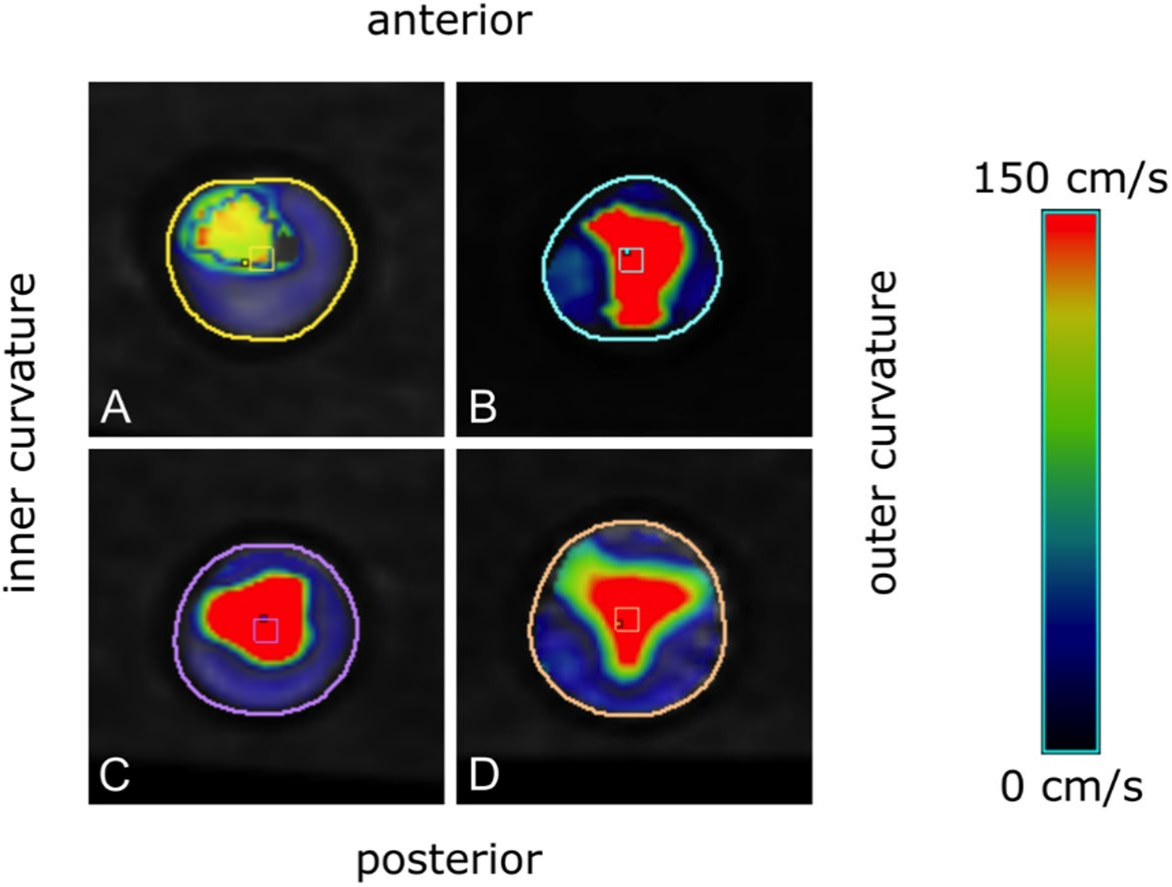
Velocity visualization within a cross-sectional plane at the *vena contracta* in 4D flow Magnetic Resonance Imaging indicating the shape of the effective orifice area: (**A**) INTUITY Elite 23 mm; (**B**) Perceval S; (**C**) SAPIEN 3 Ultra 23 mm; (**D**) Evolut PRO + 26 mm.

Figure 6 shows the qualitative shape of the EOA within the cross-sectional plane at the *vena contracta*. The INTUITY 23 mm valve had a round jet shifted towards the anterior and medial portion of the aortic wall circumference. The shape of the Perceval S was elongated, stretching from the center to the posterior wall. The jets for the remaining valves were mildly eccentric but markedly different in shape. The appearance of the jet of the two valves used in TAVR was triangular with the jet of the SAPIEN 3 Ultra being slightly shifted anteriorly and medially, and the Evolut PRO + jet being the most regular one, only slightly shifted anteriorly.

EOA estimations obtained from 4D flow MRI ranged from 2.02 to 2.24 cm^2^ in the Perceval S and INTUITY 23 mm valves. The SAPIEN 3 Ultra valve exhibited the smallest EOA of 1.71 cm^2^, while the Evolut PRO+ produced a markedly higher EOA of 2.46 cm^2^.

### Flow patterns

**Fig. 7:**
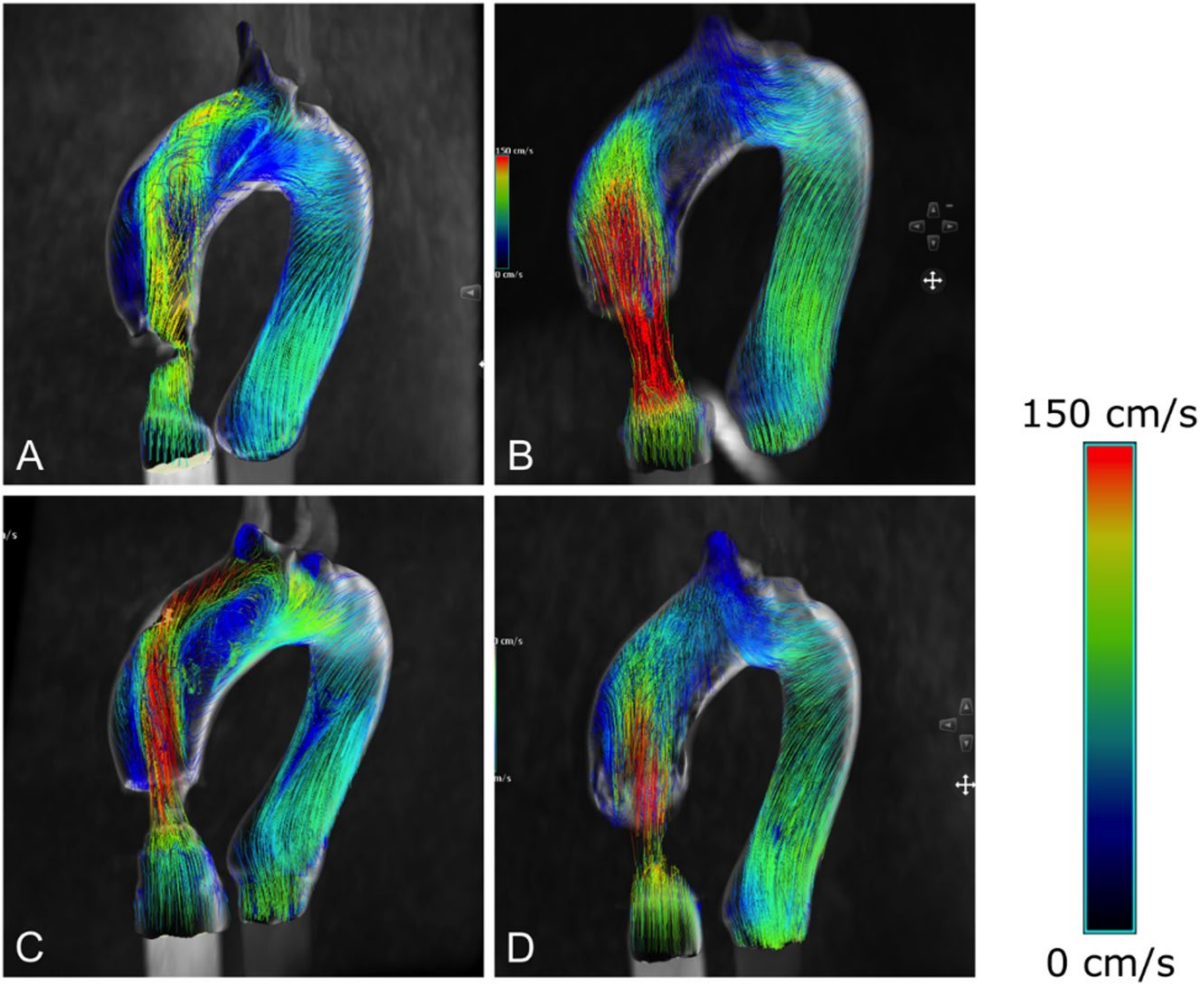
Blood flow patterns within the thoracic aorta visualized with streamlines in anterolateral view using 4D flow Magnetic Resonance Imaging: (**A**) INTUITY Elite 23 mm; (**B**) Perceval S; (**C**) SAPIEN 3 Ultra 23 mm; (**D**) Evolut PRO + 26 mm.

Qualitative assessment and comparison of flow patterns were achieved by visualizing the streamlines within the models (Fig. 7). Consistent with EOA estimations, the SAPIEN 3 Ultra valve revealed the most centric flow and most prominent flow impingement on the outer curvature of the midAoAsc and disAoAsc. Flow for the Evolut PRO + was also centric but lower in magnitude compared to the other TAVR valve. For the SAPIEN 3 Ultra a prominent clockwise swirling flow emerged from the disAoAsc. With the notable flow eccentricity already indicated by visualization of the *vena contracta* cross-section, the flow pattern associated with the INTUITY 23 mm valve was markedly different from the other valves investigated. As the forward flow dragged along the anterior vessel wall, velocity decreased while simultaneously causing secondary, irregular flows. Flow velocity along the posterior section of the midAoAsc and disAoAsc was relatively low. These qualitative observations were also consistent with the quantitative V flow US measurements revealing a notable reduction in flow velocity from the STJ to the midAoAsc within the posterior and anterior near-wall ROI.

## Discussion

The present study focusses on prosthesis-associated aortic hemodynamics in a small annulus. Investigating fluid dynamic parameters of four state-of-the-art heart valve prostheses incorporated into the same aortic arch phantom, we identified the main findings as follows:

1. The modularity of the experimental setup allows the analysis of individual patient characteristics and yields the potential to provide the necessary information to develop a simulation for the choice of heart valve prostheses.
2. The two TAVR prostheses present fundamentally different fluid-dynamic performances, despite their suggested deployment within similar sized annuli.
3. The hemodynamics of the considered TAVR valves differ from those of the two RDAVR valves mainly due to higher flow velocities and a triangular shape of the EOA.
4. The intra-annular leaflet design did not compromise hemodynamic performance in TAVR prostheses.

### Flow velocity

The obtained flow velocities using V flow US and 4D flow MRI are consistent with values determined using in vivo echocardiography after TAVR and therefor validate the experimental setup^26,27^. Average peak velocities obtained by clinical post-procedural echocardiography of the Evolut PRO 26 mm prosthesis (Medtronic) were reported at 1.7 ± 0.4 m/s^28^. A similar systematic difference between flow rates within the midAoAsc determined for the SAPIEN 3 Ultra and Evolut PRO+ prosthesis using 4D flow software was also apparent in this study. This observation did not hold for maximum flow velocities obtained by V flow US for the two TAVR prosthesis considered, with only slight differences in flow rates measured at the STJ and the midAoAsc, respectively.

Reported maximum flow velocity for stented prostheses including label-sizes of 21 to 27 mm was 2.45 m/s within the AscAo and therefore markedly higher compared to values obtained for the RDAVR prosthesis considered in the presented study, with the highest value measured at 1.87 m/s for the INTUITY 23 mm at the disAoAsc and 1.64 m/s for the Perceval S at the midAoAsc (Supplement 5)^29^.

Flow velocities obtained for TAVR prostheses are consistent with values reported by in vitro studies using flow loop setups and deploying 4D flow MRI in combination with color Doppler US. For a Portico 27 mm (Abbott Laboratories, Irvine, USA) prosthesis, maximum velocities were found at 1.7 m/s in US and about 18% higher at 2.0 m/s in MRI within the AoAsc^30^. While the herein presented, averaged flow velocity for the Evolut PRO+ prosthesis based on V flow US measurements at the STJ was also lower compared to 4D flow MRI analysis, velocities at the midAoAsc and disAoAsc were higher for US compared to MRI, resulting in an overall deviation of only 10.29% within the same section considered in the above-mentioned study.

### Wall shear stress

By means of 4D flow MRI, Farag et al. examined 14 patients after TAVR with SAPIEN 3 23 mm and 26 prostheses and found elevated WSS in the AoAsc. Mean WSS and maximum WSS were measured at 0.36 ± 0.54 and 0.90 ± 0.25 Pa, compared to 0.24 ± 0.09 and 0.62 ± 0.33 Pa in healthy control groups^31^. The WSS assessed using 4D flow MRI in the presented study was comparable to WSS measured in the healthy control, with highest mean WSS not exceeding 0.54 Pa at the STJ, and the lowest mean WSS recorded at 0.22 Pa at the midAoAsc. The same observation applies when comparing the presented findings with two in vivo studies analyzing aortic hemodynamics of healthy subjects, also including patients with bicuspid AV pathology^32,33^.

The highest WSS_Max_ at the STJ is also located at the posterior ROI for the SAPIEN 3 Ultra and the posterior and lateral (i.e. outer curvature) ROI for the Evolut PRO+ prosthesis. Notable differences can be identified for the planes positioned at the midAoAsc and disAoAsc just proximal to the brachiocephalic trunk. The WSS obtained from V flow US is markedly higher within the ROI positioned at the inner curvature (i.e. medial) and the posterior vessel wall. This trend could be observed for all TAVR and RDAVR prostheses considered, although contradictory to what would be expected from WSS visualization in 4D flow software.

### Effective orifice area, kinetic energy loss and pressure gradients

Comparing both RDAVR prostheses, the larger estimated EOA for the INTUITY 23 mm was paralleled by a lower pressure gradient. Of note, the portion of the total EL up to the STJ, i.e. relative kinetic EL, was more than twice as high for the Perceval S compared to the INTUITY 23 mm, paralleled by a markedly higher absolute kinetic EL. These results would further promote the general assumption of a larger EOA being associated with a lower kinetic EL. This observation, however, does not apply for the TAVR valves considered, as the estimated EOA of the Evolut PRO+ valve is almost 45% bigger than the EOA of the SAPIEN 3 Ultra valve, yet the relative kinetic EL determined at the STJ using 4D flow software is about 22% higher.

This discrepancy in kinetic EL is contrary to expectations based on the prostheses’ label-sizes and underlying design features. The Evolut PRO+ prosthesis has a supra-annular design, which positions the valve leaflets above the native annulus, typically providing a larger EOA, while the SAPIEN 3 Ultra has an intra-annular design, with the leaflets positioned within the native annulus^34^. While the latter provides good sealing, the EOA might be smaller, which could potentially lead to higher transvalvular pressure gradients (TPG) and increased turbulent flow, and in turn, increased kinetic EL. This assumption was also supported by a previous experimental study, where TPG measured by means of Doppler echocardiography and catheter sensors for 23 mm SAPIEN 3 and 26 mm Evolut PRO devices showed systemically higher values for the pressure gradient between the reference plane and the analysis plane positioned at the STJ, i.e. TPG^35^.

Noteworthy, TPG determined by 4D flow MRI is higher for larger label sized TAVR compared to smaller label sized prosthesis suggesting superior hemodynamic performance and less resistance to flow through the SAPIEN 3 Ultra in this patient-specific aortic model. TPG calculated by 4D flow software in the herein presented study were markedly lower compared to mean and peak TPG previously reported in two prospective multicenter studies investigating one-year outcomes after RDAVR using INTUITY and Perceval prostheses, respectively^36,37^. Contrary to expectations, the larger labelled diameter RDAVR prostheses were again associated with higher TPG. Furthermore, data suggests that the sutureless Perceval prosthesis’ supra-annular leaflet design only provided a small hemodynamic benefit over the self-expanding INTUITY models, for the larger label sizes considered.

## Limitations

The flexible material used to additively manufacture the aortic phantoms did not completely represent the biomechanical behavior of the native aorta. Despite the downstream approximation of the Windkessel effect, the fixed wall thickness and the material’s linear elastic behavior potentially led to slight alterations in the aorta hemodynamics.

While all examinations resulted in diagnostic image quality, flow within the supra-aortic vessel could not be captured. Also, local artifacts occurred in the proximity of the prosthetic stents similarly for all TAVR and RDAVR models. The reference plane and the most proximal analysis plane were therefore positioned downstream of the valve and at the level of the STJ to warrant sufficient distance from the artifact. Qualitative determination of the geometric shape of the EOA was performed by visualizing the velocity field and the plane suggested to coincide with the vena contracta. However, this method does not yield the true shape of the EOA.

## Conclusion

These findings emphasize the need for standardization of size labelling of available AV prostheses and the incorporation of hemodynamic parameters into decision-making. For the study’s patient-specific aortic phantom, the hemodynamic analysis suggests a superior performance of the INTUITY Elite 23 mm compared to the Perceval S and TAVR prostheses, also considering the conflicting interrelation of EOA, TPG, and kinetic EL of the latter. Qualitative and quantitative analysis and comparison of prostheses, as performed in the presented study using state-of-the-art cardiovascular imaging methods, could contribute to standardization. This in turn could reduce the risk of PPM and provide information on possible adverse hemodynamic effects. A better understanding of the behavior of available aortic valve prostheses in vitro will help to provide individual decision-making support for patient-specific pathologies at the time of therapy planning.

## Supplementary Information

Below is the link to the electronic supplementary material.

Supplementary Material 1

## Data Availability

The datasets used and analyzed during the current study are available from the corresponding author on reasonable request.
