# Supplementary material for: Analysis of aortic valve prostheses using advanced cardiovascular imaging—a patient-specific reversed translational approach
Source: Sci Rep. 2026 Mar 18;16:9334. doi: 10.1038/s41598-026-44295-w (PMC13002909; doi:10.1038/s41598-026-44295-w)
Supplement: Supplementary file 1 — Supplementary Material 1 [file 41598_2026_44295_MOESM1_ESM.docx]

Supplementary Material


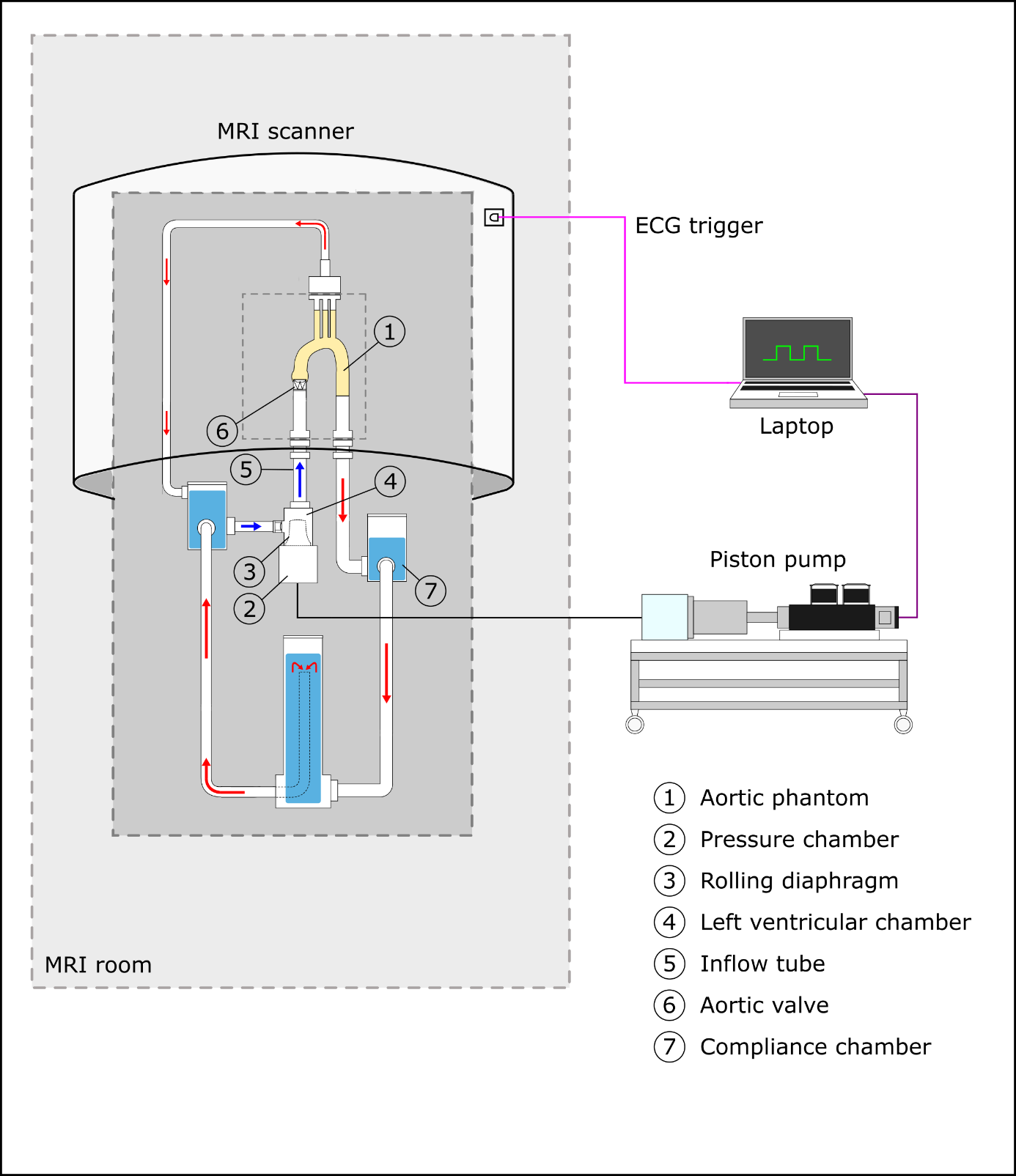


**Supplement 1**: Schematic illustration of the flow loop system placed inside the magnetic resonance imaging (MRI) scanner: blue and red arrows indicate the direction of the simulated flow; the computer and the pulsatile pump are placed outside the MRI room; the electrocardiogram (ECG) trigger cable and the pneumatic hose is guided through an MRI room’s port to the connection adapter screwed onto the pressure chamber.

**
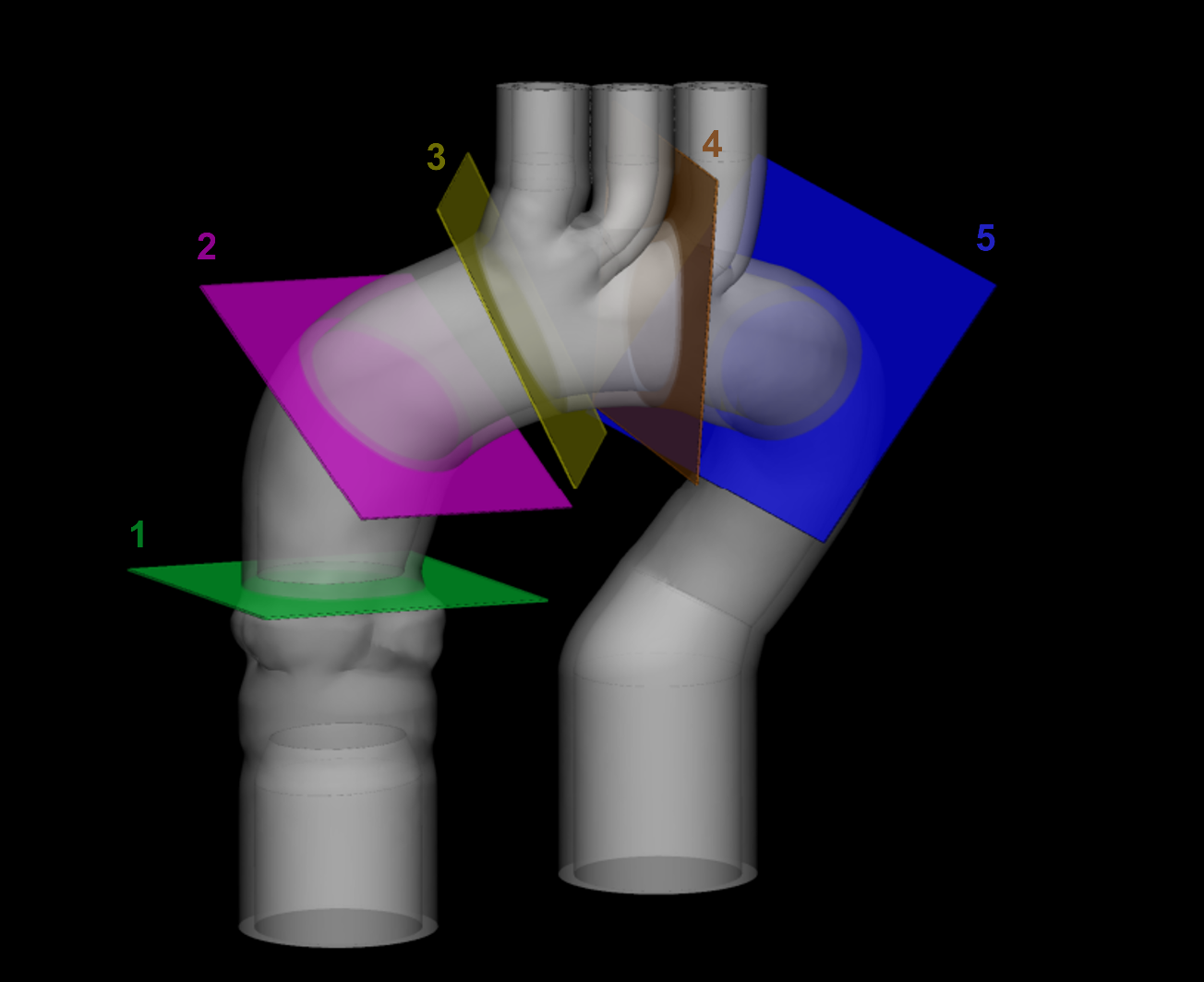
**

Supplement 2: Analysis planes defined for radiological imaging acquisition and quantitative evaluation of flow parameters with planes 1 to 3 used in Vector flow ultrasound mode and planes 1 to 5 used in 4D flow Magnetic Resonance Imaging analysis. 1 = sinotubular junction; 2 = mid-ascending aorta; 3 = distal ascending aorta; 4 = aortic arch; 5 = mid-descending aorta.

Supplement 3: Maximum wall shear stress within defined measurement planes perpendicular to the vessel’s centerline determined using 4D flow Magnetic Resonance Imaging.

|  | INTUITY Elite 23 mm | Perceval S | SAPIEN 3 Ultra 23 mm | Evolut PRO+ 26 mm |
| --- | --- | --- | --- | --- |
| STJ | 0.22 Pa | 0.35 Pa | 0.54 Pa | 0.18 Pa |
| midAoAsc | 0.17 Pa | 0.25 Pa | 0.22 Pa | 0.16 Pa |
| disAoAsc | 0.24 Pa | 0.23 Pa | 0.31 Pa | 0.17 Pa |
| AoArc | 0.24 Pa | 0.30 Pa | 0.28 Pa | 0.29 Pa |
| midAoDesc | 0.34 Pa | 0.31 Pa | 0.32 Pa | 0.25 Pa |

STJ = sinotubular junction; midAoAsc = mid-ascending aorta; disAoAsc = distal ascending aorta; AoArc = aortic arch; midAoDesc = mid-descending aorta.

Supplement 4: Kinetic EL given in µW/mm^3^ with respect to the reference plane at defined measurement planes perpendicular to the vessel’s centerline determined using 4D flow Magnetic Resonance Imaging.

|  | INTUITY Elite 23 mm | Perceva S | SAPIEN 3 Ultra 23 mm | Evolut PRO+ 26 mm |
| --- | --- | --- | --- | --- |
| STJ | 1.85 | 7.71 | 2.72 | 3.40 |
| midAoAsc | 5.76 | 17.20 | 9.85 | 7.48 |
| disAoAsc | 9.45 | 19.64 | 14.04 | 8.39 |
| AoArc | 11.64 | 22.21 | 18.35 | 8.82 |
| midAoDesc | 12.95 | 22.86 | 19.43 | 9.44 |

STJ = sinotubular junction; midAoAsc = mid-ascending aorta; disAoAsc = distal ascending aorta; AoArc = aortic arch; midAoDesc = mid-descending aorta.

Supplement 5: Flow velocities given in mm/s at defined measurement planes perpendicular to the vessel’s centerline determined using 4D flow Magnetic Resonance Imaging.

|  | INTUITY Elite 23 mm | Perceva S | SAPIEN 3 Ultra 23 mm | Evolut PRO+ 26 mm |
| --- | --- | --- | --- | --- |
| STJ | 202.18 | 254.29 | 216.38 | 190.22 |
| midAoAsc | 186.74 | 156.00 | 222.35 | 124.24 |
| disAoAsc | 180.61 | 164.04 | 262.65 | 108.32 |
| AoArc | 114.63 | 134.06 | 145.69 | 94.41 |
| midAoDesc | 172.16 | 148.93 | 162.87 | 149.76 |

STJ = sinotubular junction; midAoAsc = mid-ascending aorta; disAoAsc = distal ascending aorta; AoArc = aortic arch; midAoDesc = mid-descending aorta.
